# Neuromodulation and memory: exploring ethical ramifications in memory modification treatment via implantable neurotechnologies

**DOI:** 10.3389/fpsyg.2023.1282634

**Published:** 2023-12-21

**Authors:** Claudia González-Márquez

**Affiliations:** School of Law, The University of Edinburgh, Edinburgh, United Kingdom

**Keywords:** neuroethics, brain-computer interfaces, memory, neuromodulation, neurotechnology, justice, neuroscience

## Abstract

Invasive implantable neurotechnologies capable of simultaneously altering and recording neural activity are no longer the exclusive province of science fiction but a looming reality that will revolutionize medical practice. These advancements, particularly in their memory-altering capabilities, herald a vast array of opportunities for addressing the complex landscape of neurodegenerative and psychiatric conditions linked to memory impairments. However, the panoply of ethical implications arising from such a novel neurotechnology remains relatively unexplored by the neuroethics literature. This study examines and contrasts the potential ethical implications of memory modification treatment via implantable neurotechnologies. The study contends that undesired side effects resulting from memory modulation can lead to significant identity harms, disrupting the coherence of self-narratives and impinging on our authenticity. To evince the practical impact of this moral argument, the study conducts a practical ethical assessment of how employing implantable neurotechnologies to modulate memory may jeopardize (i) our moral responsiveness to events and core system of values and (ii) the emotional component associated with the altered memory. From a first-person standpoint, changes to the way we reasonably feel and react to past events and future intentions may be deemed ethically problematic as these profound changes can yield significant moral disruptions and negatively impact our personal lives and interpersonal relationships. In addition, the study discusses further ethical conundrums from a third-person perspective as these disruptions can inhibit social activism against structural injustices, thereby hindering societal progress. Thus, taking into account this societal dimension is paramount when evaluating the ethical permissibility of memory modification procedures.

## 1 Introduction

Implantable neurotechnologies and cutting-edge developments in the field of memory neuromodulation present new promising avenues for treating severe cognitive and neurological impairments. However, the panoply of ethical implications arising from such a novel invasive neuromodulatory context remains relatively unexplored by the neuroethics literature. The central aim of this study is to explore, highlight, and contrast the potential ethical implications of employing implantable neurotechnologies for memory modification.

This study argues that side effects arising from memory modification treatment (“MMT”) can entail significant identity harm by disrupting the coherence of our self-narratives and impinging our authenticity. To evince the practical impact of such a moral argument, the study contends that employing modulating memory may alter our moral and emotional responsiveness and the core value system. This study argues that changes to how we reasonably feel and react to past events and future intentions are ethically problematic as they can yield significant moral disruptions. The study discusses that such disruption also entails further ethical conundrums, from a third-person perspective, as it can generate a problematic collateral effect: rendering systemic change more difficult to attain. The study introduces a hypothetical case study to illustrate these issues and analyzes these potential moral harms, through a narrative lens, to personal identity. Such a perspective sheds light on ethical issues that have not been fully explored in previous identity theories and highlights the significance of the social context. The study concludes that the societal dimension must be taken into consideration when assessing the ethical permissibility of MMT.

## 2 Current debate

Drawing from the neuroethics literature on memory modification (understood as the technique of altering neural networks underlying memory), the scholarship has primarily focused on addressing ethical issues stemming from non-invasive techniques that have rendered limited success, such as transcranial magnetic stimulation or pharmacological interventions such as propranolol, chiefly due to their lack of spatial resolution and susceptibility to noise in comparison to implanted electrodes (Carter et al., 2011; Kraemer, 2013; Hui and Fisher, 2015; Racine and Affleck, 2016; Gilbert et al., 2017, 2021a; Siegel et al., 2017; Zuk et al., 2018; Cutsuridis, 2019; Klein et al., 2022). However, recent developments in the field of cognitive neuroscience pave a new horizon of neural stimulation possibilities through novel forms of memory modulation such as optogenetics, molecular modification, and implantable neural interfaces, which may overcome current limitations in non-invasive alternatives by offering higher focality and reaching deeper brain areas (Hui and Fisher, 2015; Erler, 2021; Soekadar et al., 2023). These groundbreaking prospects hold promise for patients with memory dysfunctions as they may allow us to therapeutically intervene in unprecedented ways that cannot be achieved with non-invasive means.

Among the novel memory-modifying technologies, invasive brain–computer interfaces (“iBCIs”) that target site-specific neurons through direct contact with neuronal tissue can manipulate neural circuits at high temporal and spatial resolution through, for example, ‘electrical neuromodulation’––a stimulation mechanism of electrical neural signaling––for, in this case, altering memory (Kozai et al., 2014; Ponce, 2014; Glannon, 2017; Opris, 2017; Gulino et al., 2019). For instance, a distinguishing feature of iBCIs is the possibility of *selectively deactivating* or dampening the emotional impact of undesired memories by inhibiting selected groups of neurons (Kolber, 2008; Han et al., 2009; Adamczyk and Zawadzki, 2020; Costanzi et al., 2021; Mihailov et al., 2021). Such a prospect of intentionally controlling our forgetting by modulating the stabilization process of a memory trace could erase the sting of bad memories or lessen their emotional intensity at will (Huff et al., 2013; Adamczyk and Zawadzki, 2020; Blitz and Barfield, 2023; Zawadzki, 2023).

Remarkably, ample empirical findings support this theoretical proposal. For instance, research conducted in rodent models and non-human primates has proven effective in deactivating well-consolidated memories and reducing the emotional intensity (or valence) of specific episodes (Kindt et al., 2009; Goshen et al., 2011; Lavazza, 2018; El-Shamayleh and Horwitz, 2019; Adamczyk and Zawadzki, 2020; Mihailov et al., 2021; Li et al., 2022). It should be emphasized that this prospect of advanced memory modulation in humans remains theoretical, primarily due to the ongoing experimental and clinical status of iBCIs.

In contrast to non-invasive alternatives, iBCIs pose more intricate ethical dilemmas, owing to their capacity to exploit advanced degrees of memory modification related to deliberate and selective alterations (Zawadzki, 2023), distinguishing them from other memory-modifying technologies, such as deep-brain stimulation and drugs, which lack temporal precision and operate non-selectively (van Duuren et al., 2007; Bazaka and Jacob, 2013; Trimper et al., 2018; Howell and McIntyre, 2021; Riva et al., 2021).

In such respect, Gilbert et al. (2021b) identified potential ethical risks with memory modification in human subjects. For example, there is a reasonable risk that these interventions may result in undesired and unforeseeable side effects when targeting memory formation. Notable empirical studies indicate that targeted interference of well-consolidated memories can induce abrupt neural circuit changes, including weakening the emotional component of a memory held for the subject (Redondo et al., 2014). Given memory’s associative nature, selective memory modification poses a risk of adversely altering untargeted memory chains in similar domains, causing unwanted emotional and behavioral changes (Ramirez et al., 2013; Hui and Fisher, 2015; Hu et al., 2018).

While there is a prevailing concern that neuromodulation technologies could be employed to modify fundamental human value (Aplin and Fridman, 2019; Goering et al., 2021), the ethical aftermaths arising from unforeseen side effects remain relatively unexplored from a practical perspective that considers both potential individual and societal ramifications. As the memory-modifying potential at stake is latent (Won et al., 2020; Mihailov et al., 2021; Meyer and Benoit, 2022), it calls for ethical attention in order to safeguard a responsible development without hindering their promising therapeutic potential (Zawadzki and Adamczyk, 2021a,b). To illustrate these points, the study will now introduce a hypothetical case study.

## 3 Case study

For years, Claude grappled with a haunting memory of a traumatic childhood event and developed PTSD. This traumatic episode drove him to embark on a philanthropic career path, culminating in his founding of a local fund dedicated to aiding abuse victims. After consulting with his medical team, Claude learned that an iBCI could treat his PTSD by “erasing” specific memories. He underwent the MMT in an attempt to alleviate the persistent emotional distress associated with his trauma. The procedure was deemed successful, relieving him of the psychological burden. However, in the aftermath of the procedure, Claude began to experience a profound shift in his sense of self. The philanthropic career he had pursued with unwavering commitment was suddenly seen through a different lens as the dedication-driving memory was now absent. He began contemplating leaving his local job to explore more lucrative opportunities.

## 4 Correlation between memories and self-narratives

As memory plays a building block in one’s self-narrative (Lavallee et al., 2019; Blagov et al., 2022), neuroethics research suggests that altering memories could prompt a range of psychological disruptions that may impinge upon an agent’s narrative identity (Sutin and Stockdale, 2011; Singer et al., 2013; Gilbert and Viaña, 2018). The ‘first-personal aspects of memories,’ as acknowledged by Schechtman (2011), are paramount for the process of narrative construction as they shape *who* we are, which in turn dictates *how* we are more likely to feel, act, and react toward future events. Lavazza (2019) sustains such a dyad by accentuating the intrinsic parallel memories have as part of the construction of personal identity. Thus, accessing, reflecting upon, and interpreting our memories aids our narratives to become a progressive acquisition of purpose and meaning. Through such a recalling and evaluative process, we contextualize and justify the impact our embodied experiences have had on our past and set patterns for future intentions (Postan, 2021; Leuenberger, 2021a). This brief examination regarding the entangled nexus between our memories and self-narratives will now serve to begin discussing why abrupt changes brought about by MMT are ethically alarming.

## 5 Practical ethical issues from altering memories

### 5.1 Altering moral and emotional responsiveness: first-person concerns

A current collective concern highlighted in recent neuroethics debates is the risk that the incipient memory-modifying potential of iBCIs may be used to distort emotions or absolve emotions such as blame or guilt (Forooshani et al., 2021). These changes call for ethical scrutiny as they could cause a significant shift in an agent’s core value system. Touching upon the relationship between memory and emotional response is critical for understanding these ethical issues.

Empirical evidence theorizes that specific inhibitory networks of neurons may be responsible for the intentional forgetting of memories (Ten Oever et al., 2021). Therefore, it is plausible that these networks could be key in dampening emotional responsiveness and, consequently, behavioral habituation (Barron et al., 2017; Koolschijn et al., 2019). In simple terms, modulating memory could make individuals feel and react differently than they otherwise would as their emotional responses to stimuli associated with the altered memory become distorted. As Glannon (2010) contends, memory plays a fundamental role in personal identity and moral sensibility due to the emotions it generates. As moral agents, individuals are guided by their moral emotions, and memory serves as a vital component in shaping these emotions (Liao and Sandberg, 2008; Lavazza, 2015). However, altering memory, as seen in the case of Claude, may inadvertently erode a significant portion of an individual’s moral and emotional sensitivity, potentially detaching them from their capacity to feel and respond to their own moral emotions (Erler, 2011). This complex interplay between memory and emotion inexorably raises questions of moral responsibility as, for example, the degree of ethical permissibility may vary between that of a victim or the perpetrator of a horrific act even if the act generated PTSD for both individuals (De Marco, 2019).

Neuromodulation of unpleasant memories resulting in a subsequent loss of our moral or emotional responsiveness could yield serious repercussions that will likely impair us from assessing ‘moral reasons for or against’ avenues of action as *feeling* our emotions contributes to such a counterfactual capacity of reasoning (Strawson, 1962; Rafetseder and Perner, 2012; Wilkinson et al., 2015; Glannon, 2019). For example, recalling Claude’s case, in which the original memory motivated his career to become philanthropic, it could be argued that his MMT prompted a radical emotional change that led him to disassociate his feeling of fulfillment toward his professional career. Let us examine why such an abrupt change is ethically troublesome.

Prior to the MMT, Claude’s self-characteristics developed in hand with his identity-constituting narrative, in which the memory in question held a building block. His self-narrative was coherent in light of his intelligible self-characteristics as Claude could provide an evaluative interpretation regarding *what* made him pursue philanthropy. Moreover, it could be claimed that prior to the procedure, Claude’s core value system was normative as per his identity as it constituted sufficient reasons for action (Mackenzie and Walker, 2015). Furthermore, Claude was exercising his autonomy as he was taking an active role in authoring his narrative identity––his career could be considered an authentic project of self-creation arising from his coherent self-narrative.

However, as a result of the MMT, Claude cannot provide evaluative judgment as to why he desired to support his local community. The side effects from the procedure directly impinge upon Claude’s self-narrative as the latter will now contain a significant inconsistency that will translate into a partial loss of identity (Dings and Newen, 2021). In other words, the treatment decreased Claude’s narrative coherence, and his sudden change in career could not be considered an authentic project of self-creation as such a choice would not be grounded in his long-standing values. Claude would not be able to make immediate practical sense of *who he is*, at least in his professional sphere. In Mackenzie and Walker’s (2015) terminology, Claude would not be able to make psychological and evaluative sense of himself: he would likely experience a degree of self-alienation—a likely scenario suggested in the literature (Leuenberger, 2021a,b)—as to why he is closing his charity and suddenly finds himself applying to more lucrative, non-local jobs. Though there could be external deposits of memory, such as diaries or social media publications, that could potentially help Claude weave together aspects of his narrative, if Claude is still unable to make practical sense of a crucial part of himself, this could be considered significant identity harm as such inability is breaching ‘the most basic of the capacities underpinned by coherent self-narratives’ as sustained by Postan (2022).

Claude’s case has both similarities and differences when compared to instances of the ‘burden of normality’ syndrome (“BoN”), particularly in cases where individuals with Parkinson’s disease decide to shift careers following motor improvement. A prominent similarity is that both generate an impact on the individual’s identity (Gilbert, 2012). However, the catalyst of Claude’s career change was the selective erasure of a specific memory, which led to a reevaluation of his core values. In contrast, BoN resulting in a career shift is a consequence of regaining physical abilities rather than a direct consequence of memory alteration. BoN is often perceived as a restoration of previous capabilities before the onset of the condition––authenticity is maintained, and a career shift is a result of returning to a more familiar state.

On the other hand, MMT directly involves altering an individual’s emotional experiences––a more profound change in the realm of personal identity because it directly affects the way a person perceives and interacts with their past experiences and values. Even though memory is extended and socially constructed (Hunt, 2013), it is closely tied to one’s sense of self and moral sensibility; thus, its alteration can be regarded as a more fundamental interference than regaining physical abilities.

In addition, Claude’s identity harm also entails further repercussions from a third-person perspective. For instance, his abrupt lack of career commitment could be perceived as selfish by others as Claude’s colleagues would not comprehend his sudden decision to close the organization that he dedicated decades of effort to building. Additionally, the change could also bear repercussions on his family life; for example, Claude’s wife would not understand why he wants to move countries and might not be able to carry on a long-distance marriage. This scenario evinces the potential negative consequences of altering memory, particularly the significant harm to our personal lives and interpersonal relationships.

Claude’s case is contentious as some may argue that if he intentionally altered his memory, his decision could be considered a narrative development that he autonomously constructed (Wiley et al., 1998; Rocha, 2014). However, as put forward by Tan and Lim (2020), a forceful alteration of one’s self-narrative can also be inherently self-deceiving. In such a line of argument, Claude’s choice of memory editing could not be considered authentic as it would not exhibit a sufficient degree of intelligibility as per his pre-existing narrative (Libby et al., 2011).

### 5.2 Altering the emotional component of memories: social considerations

The possibility of emotional blunting brings the discussion to a larger issue that calls for ethical scrutiny in its own right. This section will examine how MMT can entail an ethically problematic collateral effect: rendering systemic change more difficult to attain. The aim of this section is two-fold. First, it is argued that societal considerations can multiply the conflicting moral views regarding whether MMT is ethically justified. Second, ethical implications stemming from MMT shall be examined in relation to concerns on an individual level and amalgamated with potential societal issues as narratives are inescapably socially embedded.

It is relevant to note that the ethical implications of MMT are contingent on distinguishing between particular memory erasure and emotional blunting. While Claude’s case primarily involved the erasure of a specific traumatic memory, MMT can encompass various forms of memory modulation, including preventing the formation of new traumatic memories and limiting the ability to recall past experiences. Thus, the implications arising from MMT depend on the specific nature of the intervention. Erasing traumatic episodes raises concerns related to authenticity and potential identity harm. In contrast, emotional blunting or preventing the formation of new traumatic memories also carries distinct ethical concerns, potentially reducing empathy and hindering an individual’s capacity to relate to the experiences of victims of trauma, impeding advocacy for social change.

The emotional component of our memories (particularly traumatic episodes) plays a crucial role in one’s narrative construction process as it constitutes an aggregate factor for providing explanatory justification for our lived experiences. However, such a component is also crucial for instituting social change. Tenenbaum and Reese (2007) sustain the latter by arguing that memories provide a foundation for systemic changes. Moreover, Gensburger (2020) contends that shared traumatic memories of, for example, violence are relevant in order to collectively aim to pacify societies, combat racial bias, and lessen discrimination rates.

In such respect, the President’s Council on Bioethics (Beyond Therapy, 2003) has argued that memories provide us with invaluable information that aids us in re-evaluating our social institutions. In virtue of this, social movements such as *#MeToo* or *#BlackLivesMatter* have demonstrated that intersubjective, collective types of traumas serve as an emotional stimulus and encourage individuals to fight systemic issues that they have experienced, such as racism, violence, or sexual abuse.

As echoed by multiple scholars (Barry et al., 2020; Forooshani et al., 2021), the capacity to *feel* the emotions associated with particular memories is a paramount driver for fostering social change; striving for social change demands––in Nezu et al. (2021) terminology––a cognitive openness to negative emotions. Thus, it is rational to assert that the emotional component in question serves to advocate for systemic change by acting as a catalyst for fighting social injustices. Translating this argument into our neuromodulatory context, dampening memories can also entail a significant risk of making us unable to decolonize existing systemic issues as feeling the emotions correlating to our memories is crucial for achieving the former.

Hence, from a third-person standpoint, blunting memories’ emotional component carries an added ethical dilemma as such alteration can generate significant barriers to attaining *justice*. Let us illustrate the practical ethical impact of this contention by recalling Claude’s case. The act of closing his charity would not only constitute an inauthentic choice, but one that would hinder social change as, for example, the foundation may have played a pivotal role in tackling inequalities by providing support to abuse victims and promoting educational developments within Claude’s local community. In such a case, closing the charity would impinge upon Claude’s social context by ceasing the advocacy for valuable social change and reducing the efforts to lessen the inequities that the foundation was tackling. With this reasoning, if our ability and willingness to strive for social change––a reasonable emotional reaction after experiencing trauma––is disrupted due to MMT, then it can be argued that such a procedure could lessen our advocacy for valuable justice. Claude’s case highlights the complexity and impact between our narratives and the social world.

## 6 The moral divergence between individual values and societal interests

In circumstances when a memory entails psychophysical distress that severely deteriorates one’s quality of life, moral disagreements regarding the risks and benefits of MMT would undoubtedly arise. Some might argue that it could be in an agent’s best interests to modulate a traumatic episode albeit the latter might constitute a relevant building block of their identity that could lead the individual to live an inauthentic life and lose a fragment of his narrative (Liao and Sandberg, 2008). For example, a degree of identity harm may be ethically permissible on the grounds of *wellbeing* or a transient effect of inauthenticity might be subjectively worth the alleviation from severe mental disorders (Kostick-Quenet and Lázaro-Muñoz, 2021; Kostick-Quenet et al., 2022). However, granting ethical priority to certain values over others would not be imperative in these scenarios but would be contingent on a case-by-case basis. Certainly, the moral exercise of balancing risks and benefits will constitute a complex task due to entangled societal concerns. Thus, it is imperative to take these elements into consideration and disclose them as necessary prior to these medical procedures being undertaken. Indubitably, moral tension will subsist due to the range of moral discrepancies that these novel procedures will generate.

## 7 Conclusion

While this debate cannot be settled here, it is pragmatic to sift out and highlight the array of plausible moral divergences that would originate when ethically justifying MMT. As shown, the moral landscape surrounding memory modulation is multifaceted. Potential disruptions to values should not be the sole determinant to exclude the use of neuromodulatory technologies but rather complement the informed, decision-making process by systematically allowing individuals to better understand, acknowledge, and balance the diverse impacts on their personal and relational sphere associated with erasure, emotional blunting, or other forms of memory modulation. Within the frontier of neurotechnology regulation, the ongoing discourse on neurorights initiatives, advocating personal identity as a human right, can offer a valuable framework for addressing the ethical considerations of neuromodulatory technologies that may disrupt an individual’s sense of self.

## Data availability statement

The original contributions presented in the study are included in the article/supplementary material, further inquiries can be directed to the corresponding author.

## Author contributions

CG-M: Writing – original draft, Writing – review & editing.
